# Effect of initial soil properties on six‐year growth of 15 tree species in tropical restoration plantings

**DOI:** 10.1002/ece3.2508

**Published:** 2016-11-15

**Authors:** Cristina Martínez‐Garza, Julio Campo, Martin Ricker, Wolke Tobón

**Affiliations:** ^1^ Centro de Investigación en Biodiversidad y ConservaciónUniversidad Autónoma del Estado de MorelosCuernavacaMexico; ^2^ Instituto de EcologíaUniversidad Nacional Autónoma de MéxicoMexico CityMexico; ^3^ Instituto de BiologíaUniversidad Nacional Autónoma de MéxicoMexico CityMexico; ^4^ Comisión Nacional para el Conocimiento y Uso de la Biodiversidad (CONABIO)Mexico CityMexico

**Keywords:** Los Tuxtlas, Mexico, soil nutrients, tree performance, tropical rainforest

## Abstract

In restoration plantings in degraded pastures, initial soil nutrient status may lead to differential growth of tropical tree species with diverse life history attributes and capacity for N_2_ fixation. In 2006, we planted 1,440 seedlings of 15 native tree species in 16 fenced plots (30 × 30 m) in a 60‐year‐old pasture in Los Tuxtlas, Veracruz, Mexico, in two planting combinations. In the first year, we evaluated bulk density, pH, the concentration of organic carbon (C), total nitrogen (N), ammonia (${\text{NO}}_{3}^{-}$), nitrate (${\text{NH}}_{4}^{+}$), and total phosphorus (P) in the upper soil profile (0–20 cm in depth) of all plots. The first two axes of two principal component analyses explained more than 60% of the variation in soil variables: The axes were related to increasing bulk density, ${\text{NO}}_{3}^{-}$, ${\text{NH}}_{4}^{+}$, total N concentration, and pH. Average relative growth rates in diameter at the stem base of the juvenile trees after 6 years were higher for pioneer (45.7%) and N_2_‐fixing species (47.6%) than for nonpioneer (34.7%) and nonfixing species (36.2%). Most N_2_‐fixing species and those with the slowest growth rates did not respond to soil attributes. Tree species benefited from higher pH levels and existing litter biomass. The pioneers *Ficus yoponensis*,* Cecropia obtusifolia*, and *Heliocarpus appendiculatus*, and the N_2_‐fixing nonpioneers *Cojoba arborea*,* Inga sinacae*, and *Platymiscium dimorphandrum* were promising for forest restoration on our site, given their high growth rates.

## 1 Introduction

Large parts of the tropical rainforest in the world have been deforested for cattle ranching activities (Chazdon, 2014; Fearnside & Barbosa, 1998). Deforestation and livestock cause soil and nutrient losses due to erosion, leaching, and volatilization (Bolin & Sukumar, 2000; Neill & Davidson, 2000; Silver, Kueppers, Lugo, Ostertag, & Matzek, 2004; Steinfeld, Hann, & Black Burn, 2013). The effects of these processes on some soil cycles have been reported as positive (Garcia‐Montiel et al., 2000; Guo & Gifford, 2002), negative (Neill et al., 1995, 1997; Tobón, Martínez‐Garza, & Campo, 2011), or nonsignificant (Hughes, Kauffman, & Jaramillo, 2000). These differences in results have been related to the precipitation regime, topographic position, intensity of soil use, and the time an area has been used as pasture for cattle (Guo & Gifford, 2002; Silver et al., 2004). Soil nutrients under deforestation scenarios are influenced by the footprints of large biogeochemical heterogeneity in tropical forest soils (Townsend, Asner, & Cleveland, 2008), besides their sensitivity to environmental conditions and land‐use history.

After pastures are abandoned, natural succession toward forest can take decades, due to reduced soil fertility (Aide, Zimmerman, Herrera, Rosario, & Serrano, 1995; Myster & Pickett, 1992) and lack of propagules (Holl, 1999; Martínez‐Garza, Flores‐Palacios, De La Peña‐Domene, & Howe, 2009). Plantings of native tree species have been used as a tool to accelerate natural succession (Lamb, Erskine, & Parrotta, 2005) and carbon sequestration (Silver et al., 2004). Planting fast‐growing pioneer tree species is recommended for sites with low potential for natural recovery (Holl & Aide, 2011); however, given that nonpioneer trees determine most of the ecological processes in the old‐growth target forests (Chazdon, 2014), they are also recommended for restoration plantings (Elliot et al., 2003). Pioneers and nonpioneers are the extremes of a life history continuum: Pioneer trees have regenerative strategies associated with high‐resource availability, such as fast growth rates and short life spans of about 30 years (Swaine & Whitmore, 1988; Whitmore, 1989); they show strong responses to light quality and quantity (King, 1994; Tinoco‐Ojanguren & Pearcy, 1995) and small seeds (Foster & Janson, 1985). On the other extreme, nonpioneers show lower growth rates and large variation in light requirements (Swaine & Whitmore, 1988); they tend to have large seeds (Ibarra‐Manríquez, Martínez‐Ramos, & Oyama, 2001). The growth of tree species in pastures may be predicted to some extent from their life history attributes, as observed under old‐growth forest conditions; highly eroded soils in pasture lands, however, may lead to varying growth of selected tree species unrelated to their life history, but rather to their response to soil characteristics.

Growth of tree species in restoration plantings may be affected by initial xeric conditions of disturbed areas. Pioneer species may benefit from high light levels and tolerate high heat loads (Ceccon, Huante, & Campo, 2003; Huante, Rincon, & Chapin, 1998). Furthermore, tree species capable of symbiotic fixation (N_2_‐fixing species) may outperform nonfixing species under the high light conditions and poor soils of early‐successional environments, and therefore be less affected by low content of nutrients in the soil (Barron, Purves, & Hedin, 2010; Sprent, 2009). After several years of natural succession, changes in aboveground and belowground conditions due to plantings and natural recruitment (de la Peña‐Domene, Martínez‐Garza, & Howe, 2013; Guariguata, Rheingans, & Montagnini, 1995) may result in similar performance of species, irrespective of life history attributes (Carpenter, Nichols, & Sandi, 2004) or N_2_‐fixing capacity (Menge & Chazdon, 2016). Not only the time frame for these events under managed succession, but also the effect of initial soil properties on tree species with different life history attributes and N_2_‐fixing capacity are not known.

In this study, we evaluated the effect of initial soil properties (bulk soil density, pH, and the concentration of organic carbon [C], nitrate [ ${\text{NO}}_{3}^{-}$], ammonium [${\text{NH}}_{4}^{+}$], total nitrogen [N], and total phosphorus [P]) on five pioneer and 10 nonpioneer tropical rainforest tree species, after six years of planting in a 60‐year‐old pasture in the wet tropics of Mexico. The main question addressed was: Do tree growth rates reflect initial soil properties? Given that life history category and attributes such as seed mass and N_2_‐fixing capacity of trees have been related to growth rates, we expect these traits to be useful to predict the growth response of species to soil nutrient status. Detailed analysis of relationships between biogeochemical soil properties and tree growth may allow more confident selection of tree species by life history attributes or N_2_‐fixing capacity; this will also allow more informed decisions on soil management to increase growth rates of trees for restoration plantings or commercial plantations.

## 2 Methods

The Los Tuxtlas geographic region is a 315,525‐ha volcanic elevation at the Gulf coast of Mexico with tropical humid climate and vegetation (Gutiérrez‐García & Ricker, 2011). Research was carried out on a pasture area adjacent to the Los Tuxtlas Biological Station, a part of the Los Tuxtlas Biosphere Reserve (18°05′ and 18°45′N; 94°35′ and 95°30′W), Veracruz, Mexico. Mean annual rainfall at the station from 1997 to 2007 was 4,275 ± 404 mm, with a rainy season from June to February that concentrates 92% of the total annual precipitation; the mean annual maximum temperature was 28°C (R. Coates, National University of Mexico, Veracruz, *personal communication*). Soils at the study site were classified as entisols, from *typic ustorthents* to *lithic ustorthents* (Tobón et al., 2011). These soils originated from rocks of basalt and andesite, mixed with volcanic ash; they have a mainly clayey texture (clay content = 48.5%) and tend to be acidic (pH = 4.9) (González‐Soriano, Dirzo, & Vogt, 1997). The forest in the area is a lowland tropical rainforest with a closed canopy up to 35 m high; *Nectandra ambigens* (Lauraceae) is a common species in the canopy layer, and *Pseudolmedia glabrata* (Moraceae) and *Astrocaryum mexicanum* (Arecaceae) in the mid‐canopy and understory, respectively (Bongers, Popma, Meave‐del‐Castillo, & Carabias, 1988; Popma, Bongers, & Meave del Castillo, 1988).

The pasture site is on a hillside gradient from 182 to 260 m above sea level within a broad valley, facing NE to the Gulf of Mexico. The original forest of the site was cleared approximately 60 years ago, the remaining vegetation was burned, and corn was planted for one season, together with exotic (*Cynodon dactylon*,* Cynodon plectostachyus*,* Panicum* spp., *Urochloa brizantha,* and *Urochloa decumbens*) and native (*Axonopus compressus* and *Paspalum conjugatum*) grasses. Herbicides have been applied as often as necessary to control shrubs; due to a decrease in productivity, cattle stocking rates have decreased over 30 years from three to two cows per hectare (de la Peña‐Domene et al., 2013). Grass in unfenced pastures was a 5‐ to 10‐cm‐tall mix of exotic species and native species.

**Figure 1:**
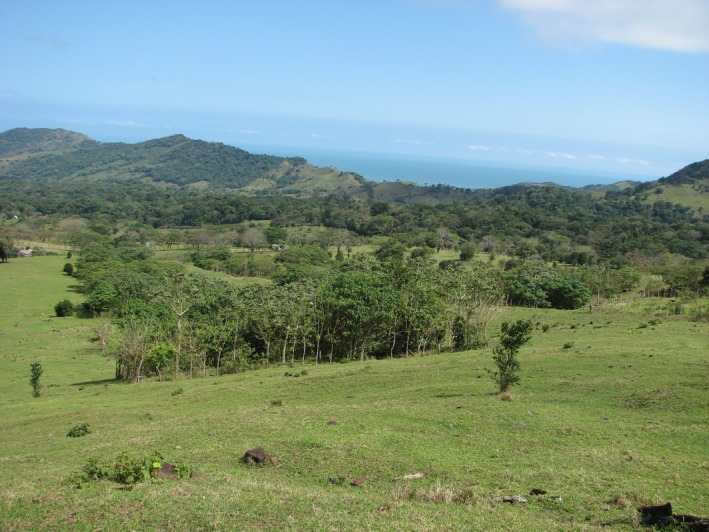
Plot with plantings at Los Tuxtlas, Veracruz, Mexico

Twenty‐four plots in a 3 × 8 grid were established in August 2006 in a 12‐ha pasture within the agricultural Colony of Ruiz Cortines. Each of the 24 plots have a size of 30 × 30 m; each plot was divided into four subplots by 3‐m corridors to establish nets for fauna census (Figure 1). Plots are separated by 35 m of active pasture, and each of them is surrounded by a 1.6‐m‐tall fence to exclude cattle. Isolated trees within the 12‐ha pasture were cut in October and November 2006 (Howe, Urincho‐Pantaleon, de la Peña‐Domene, & Martínez‐Garza, 2010).

Seedlings of 18 species were raised in a nursery from February to August 2006 at the nearby Los Tuxtlas station. Seedlings germinated in the shade in the nursery, using a 50 cm^3^ soil inoculum from the site of seed collection, to include mycorrhizae. A total of 144 seedlings were planted at a spacing distance of 2 × 2 m, with 12 seedlings per species. Seedlings were 4–7 months old at planting, with an average height of 17.8 cm, ranging from 5 to 40 cm across species. Seedlings were planted in the middle of the wet season of August 2006 (2,208 seedlings; Martínez‐Garza, Tobón, Campo, & Howe, 2013). Plantings are part of a long‐term experiment to evaluate the effects of dispersal vector of planted species in the seed rain (Martínez‐Garza et al., 2009) and recruitment (de la Peña‐Domene et al., 2013). Therefore, two combinations of plantings were established: Eight plots were originally planted with 10 animal‐dispersed tree species, from which three were pioneer and seven were nonpioneer species (mixture 1); another eight plots were planted with eight wind‐dispersed tree species, from which three were pioneer and five were nonpioneer species (mixture 2; Table 1); and eight plots remained as control plots without plantings and are not included in the analysis. Seedlings of different species within their corresponding mixture were planted in mixed stands. Classification of species as pioneers or nonpioneers was based on the literature (Ibarra‐Manríquez & Oyama, 1992; Martínez‐Ramos, 1985; Popma, Bongers, & Werger, 1992). At each planting mixture, two legumes were initially planted: *Cojoba arborea* and *Inga sinacae* (mixture 1) and *Platymiscium dimorphandrum* and *Albizia purpusii* (mixture 2). These species are included in Appendix 1 of Sprent (2009) except for *I. sinacae*; however, all the species in that genus are presumed to nodulate (Sprent, 2009). Fifteen native tree species from those originally planted in 2006, with enough surviving juveniles after 6 years were selected for this study (Table 1). Species also varied in maximum tree height (from small‐statured trees of the forest understory of about 3 m to emergent canopy trees of about 40 m), and average seed mass (Table 1). Herbaceous vegetation was removed every two months in a radius of 0.5 m around the planted trees to control grass competition (see Holl, 1999), until the planted trees were taller than the grass (about 1.5 m). No fertilizer was added to plantings. Basal stem diameter was measured annually from May 2007 to May 2013 for all trees.

**Table 1:** Family and seed mass (g) of five pioneer and 10 nonpioneer tropical tree species planted in pastures at Los Tuxtlas, Veracruz, Mexico, in two combinations (mixtures)

Species	Family	Average Seed mass
Pioneers		
*Cecropia obtusifolia* a	Cecropiaceae	0.009
*Cedrela odorata* b	Meliaceae	0.020
*Ficus yoponensis* a	Moraceae	0.001
*Heliocarpus appendiculatus* b	Tiliaceae	0.004
*Ochroma pyramidale* b	Bombacaceae	0.007
Nonpioneers		
*Amphitecna tuxtlensis* a	Bignoniaceae	0.600
*Bernoullia flammea* b	Bombacaceae	0.330
*Brosimum alicastrum* a	Moraceae	0.960
*Cojoba arborea* a	Mimosaceae	0.600
*Cordia megalantha* b	Boraginaceae	0.110
*Inga sinacae* a	Mimosaceae	0.360
*Platymiscium dimorphandrum* b	Fabaceae	0.160
*Poulsenia armata* a	Moraceae	0.085
*Pouteria sapota* a	Sapotaceae	22.50
*Tabebuia guayacan* b	Bignoniaceae	0.001

Species were assigned *a priori* to pioneer or nonpioneer groups following the literature (Ibarra‐Manríquez & Oyama, 1992; Martínez‐Ramos, 1985). Taxonomy follows Ibarra‐Manríquez and Sinaca (1995, 1996a,b).

a Mixture 1.

b Mixture 2.

The soil physical and chemical properties were evaluated from composite samples of each plot, one taken at the middle of each of four subplots from two soil depths (0–5 and 5–20 cm depth), collected in the middle of the rainy season (October) of 2006. The litter samples consisted of all dead plant material lying on the forest floor. Prior to analysis, soil samples were air‐dried and sieved (2‐mm mesh). The fine fraction was used to determine concentrations of organic C, total and mineral N (${\text{NO}}_{3}^{-}$ and ${\text{NH}}_{4}^{+}$), and total P in the soil. Soil organic C was analyzed in an automated C‐analyzer. The concentrations of total N and P in soil were determined after acid digestion of samples in concentrated H_2_SO_4_. The mineral N was extracted in 2 mol/L KCl (Robertson et al., 1999). Concentrations of soil mineral N, total N, and total P were determined with a NP elemental analyzer.

### 2.1 Data analyses

#### 2.1.1 Growth rates in diameter at the stem base

We calculated logarithmic relative growth rates of diameters at the stem base (*lnRGR*) for 6 years for each individual, using the following equation:

$$\text{InRGR}=\ln\left[\frac{\ln\left[D_{2}\right]-\ln\left[D_{1}\right]}{t_{2}-t_{1}}\right],$$

where ln is the natural logarithm, *D* refers to the diameter, *t* to a point in time, and the subindexes 1 and 2 to the beginning and the end of the measuring period. Using the natural logarithm of relative growth rate normalizes to a large extent the residuals in analysis of variance (ANOVA) (Ricker, Peña Ramírez, & von Rosen, 2014).

#### 2.1.2 Soil properties

Two principal component analyses (PCAs) were run to ordinate nine variables of soil status using the average of the two soil depths. Given that tree species were planted in two combinations, the correlations with the soil attributes are based on the soils collected in the plots where the species were planted. Subsequently, the first two axes of each of these PCAs were used to predict tree performance of species from their corresponding plot's soil characteristics; in these correlations, trees were used as replicates. Some soil data overlap with Tobón et al. (2011), who evaluated litter and soil properties in the pasture plots and closest conserved forest in three slope positions; however, they did neither analyze soil properties by plot nor its effects on growth rates.

To test the effect of initial soil properties on *lnRGR*, Pearson correlation coefficients were calculated using the first two PCA axes and average *lnRGR* separately by life history strategy (pioneers and nonpioneers) and by N_2_‐fixing capacity (N_2_‐fixing and nonfixing species). Visual inspection of scatter plots helped to identify outliers. ANOVAs and Pearson correlations were performed in STATISTICA 7.0 (StatSoft, 2004).

## 3 Results

### 3.1 Initial soil properties

Nine chemical and physical soil properties were evaluated during the first year at superficial soil depth (0–20 cm; Table 2). For PCA of mixture 1, the first two axes explained 62.3% of the variation in soil variables among the eight plots (Supplementary Material Figure S1a). The PCA axis 1 was related to increasing bulk density and ${\text{NO}}_{3}^{-}$, whereas axis 2 was related to increasing bulk density and ${\text{NH}}_{4}^{+}$. For this analysis, plot 22 showed the highest values for axis 1, related to the highest bulk density (Figure S1a).

**Table 2:** Mean, standard deviation (*SD*), minimum, maximum, and coefficient of variation (CV; %) of nine chemical and physical soil properties in (a) eight plots of mixture 1 and (b) eight plots of mixture 2 in 2006 at superficial soil depth (0–20 cm) at Los Tuxtlas, Veracruz, Mexico

	Mean ± *SD*	Min	Max	CV
(a)				
Bulk density (g/cm^3^)	0.90 ± 0.07	0.82	1.02	7.77
pH (H_2_0)	5.78 ± 0.07	5.69	5.90	1.14
Organic C (mg/g)	48.62 ± 8.61	33.54	57.54	17.70
${\text{NO}}_{3}^{-}$ (μg/g)	10.60 ± 3.19	4.41	13.88	30.13
${\text{NH}}_{4}^{+}$ (μg/g)	7.69 ± 2.61	5.01	13.46	33.92
Total N (mg/g)	4.17 ± 0.98	3.32	6.28	23.41
Total P (μg/g)	288.23 ± 68.47	174.61	387.85	23.75
Mineral N (NO^–^_3_ + NH^+^_3_)	18.29 ± 4.00	11.08	24.76	21.85
Litter dry biomass (g/m^2^)	28.83 ± 45.98	3.76	141.22	159.48
(b)				
Bulk density (g/cm^3^)	0.93 ± 0.06	0.85	1.05	6.79
pH (H_2_0)	5.79 ± 0.14	5.69	6.10	2.34
Organic C (mg/g)	43.95 ± 10.08	27.13	57.99	22.94
${\text{NO}}_{3}^{-}$ (μg/g)	11.24 ± 2.37	7.81	14.18	21.09
${\text{NH}}_{4}^{+}$ (μg/g)	7.46 ± 1.12	5.42	8.83	15.06
Total N (mg/g)	3.52 ± 0.70	2.58	4.75	20.01
Total P (μg/g)	296.25 ± 100.92	120.58	460.26	34.07
Mineral N (NO^–^_3_ + NH^+^_3_)	18.7 ± 2.01	14.88	21.21	10.77
Litter dry biomass (g/m^2^)	21.04 ± 16.76	7.14	47.66	79.66

For PCA of mixture 2, the first two axes explained 66.2% of the variation in soil variables among the eight plots (Figure S1b). The PCA axis 1 was related to increasing total N concentration and bulk density, whereas axis 2 was related to increasing ${\text{NH}}_{4}^{+}$ and pH. For this analysis, plot 3 showed the lowest values of axis 2 related to the highest values of pH (6.24) and lowest ${\text{NH}}_{4}^{+}$ (Figure S1b).

### 3.2 Relative growth rates in diameter

Logarithmic relative growth rates in diameter at the stem base (*lnRGR*) for 15 tree species after six years of growth varied up to three times among species. On average, five pioneer species had significantly higher *lnRGR* (RGR = 45.7%) than 10 nonpioneer species (RGR = 34.7%; *F*_1,89_ = 14.85, *p* < .001). The analysis of variance revealed significant differences in *lnRGR* among species (*F*_14,76_ = 16.31, *p* < .0001; Figure S2). The three N_2_‐fixing species (*C. arborea*,* I. sinacae,* and *P. dimorphandrum*) had significantly higher *lnRGR* in diameter (RGR = 48.0%) than the other 11 nonfixing species (RGR = 36.0%; *F*_1,89_ = 10.73, *p* < .001). When the analysis was run only with nonpioneer species, the N_2_‐fixing species had almost twice the *lnRGR* than the seven nonfixing species evaluated (RGR = 28.0%; *F*_1,53_ = 44.76, *p* < .0001).

Seed mass of tree species ranged from 0.001 to 22.5 g (Table 1). Including all 15 tree species, seed mass predicted significantly growth rates in linear regression analysis: Those species with smaller seeds showed higher growth rates (*lnRGR* = −1.20 to 0.08 × *ln* [seed mass], *r* ^2^ = .47, *p* < .005; Figure S3). The power of prediction increased when N_2_‐fixing species were removed (*lnRGR *= −1.36 to 0.10 × *ln* [seed mass]; *r* ^2^ = .80; *p* < .0001). Seed mass also predicted growth rates when only nonpioneer species were considered (*lnRGR* = −1.36 to 0.09 × *ln* [seed mass]; *r* ^2^ = .75; *p* < .01).

### 3.3 Effect of initial soil properties on species growth rates

**Figure 2:**
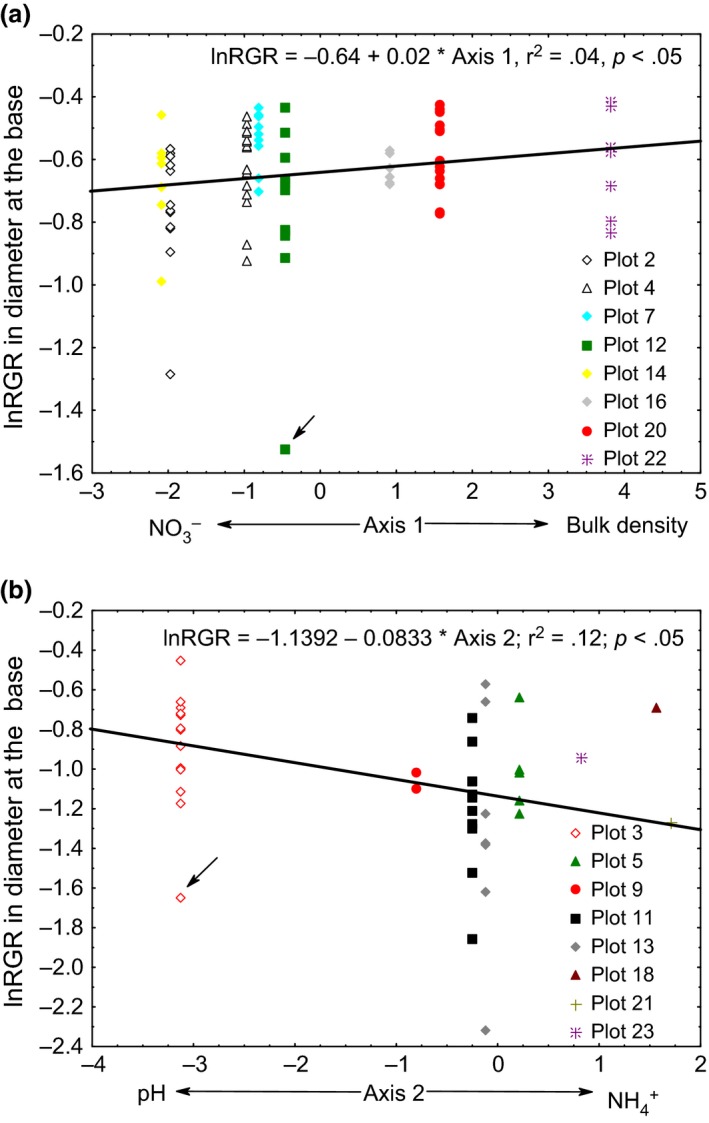
Regression of the logarithmic relative growth rate (*lnRGR*) as a function of PCA scores with soil attributes of (a) two pioneer species of mixture 1, and (b) six nonfixing species of mixture 2. Value of *r* ^2^, regression line, and equation are shown. Arrows point at an outlier not included in the regression. Plots are indicated with different symbols

Principal component analysis axis 1 was correlated with *lnRGR* for pioneer species of mixture 1 (Figure 2a ; Table 3) whereas *lnRGR* was not correlated with axis 2. The first two axes of the PCA were not correlated with the *lnRGR* for the six nonpioneer species (Table 3). The first two axes of the PCA were not correlated with *lnRGR* neither for the two N_2_‐fixing species nor for the six nonfixing species (Table 3). At the species level, the *lnRGR* of *Brosimum alicastrum* (Figure S4a) and *C. arborea* (Figure S4b) were positively correlated with axis 1 of the PCA. For the other three nonpioneer and two pioneer species, axes 1 and 2 were not correlated with lnRGR (Table S2).

**Table 3:** Pearson correlation coefficients (*r*) of PCA axes 1 and 2 and *lnRGR* for tree species by life history category and N_2_‐fixing capacity in two planting combinations in a tropical pasture at Los Tuxtlas, Veracruz, Mexico. Coefficients of determination (*r* ^2^) are shown in graphs for significant correlations

	PCA axis 1	PCA axis 2
Mixture 1		
Pioneers	.21^a^	.07
Nonpioneers	.07	−.07
N_2_ fixing	.19	−.13
Nonfixing	.07	.03
Mixture 2		
Pioneers	−.13	−.10
Nonpioneers	−.09	−.09
N_2_ fixing	−.02	−.07
Nonfixing	−.15	.35^a^

a *p *<* *.05.

The first two axes of the PCA were correlated with *lnRGR* neither for the three pioneers nor for the four nonpioneer species of mixture 2 (Table 3). The first two axes of the PCA were not correlated with *lnRGR* for the N_2_‐fixing species (Table 3). Axis 1 was not correlated to *lnRGR* for the six nonfixing species whereas axis 2 was correlated with the *lnRGR* for the six nonfixing species (Figure 2b; Table 3). At species level, the axis 1 of the PCA was negatively correlated with *lnRGR* of *Cordia megalantha* (Figure S4c) and *Tabebuia guayacan* (Figure S4d), whereas axis 2 was negatively correlated with *lnRGR* of *Heliocarpus appendiculatus* (Figure S4e) and *Ochroma pyramidale* (Figure S4f). For the other pioneer and two nonpioneer species, axes 1 and 2 were not correlated with *lnRGR* (Table S2).

## 4 Discussion

Since deforestation took place 60 years ago, cattle activities and erosion processes have changed the soil nutrient status on our pasture sites at Los Tuxtlas (Roa‐Fuentes, Martínez‐Garza, Etchevers, & Campo, 2015; Tobón et al., 2011). After six years, most N_2_‐fixing species planted, and those with the slowest growth rates did not respond to soil attributes. Tree species benefited from higher pH levels, due to the acidic reaction condition of the soil, and higher litter mass, that represents a source of nutrients for plant growth released by decomposition.

### 4.1 Initial soil properties

Soil nutrient properties of the over 60‐year‐old pasture were mainly related to variation in the concentrations of ${\text{NH}}_{4}^{+}$, ${\text{NO}}_{3}^{-}$, and total N, pH, and bulk density. Significant increases in ${\text{NH}}_{4}^{+}$ and ${\text{NO}}_{3}^{-}$ concentrations in soil of heavily grazed pastures have been registered as a consequence of deposition of cattle excrement and urine in Australia woodlands (Yates, Norton, & Hobbs, 2000), and dominated the inorganic pool of ${\text{NH}}_{4}^{+}$ in Australian tropical pastures (Paul, Catterall, Pollard, & Kanowski, 2010a). This was not the case for our tropical pasture site in Mexico: The concentration of ${\text{NH}}_{4}^{+}$ was twice as low in the grazed pasture as in the soil of the conserved forest (Table S1). On the other hand, the concentration of ${\text{NO}}_{3}^{-}$ was similar in the grazed pasture and the soils of the conserved forest (Table S1). The variation in ${\text{NH}}_{4}^{+}$ and ${\text{NO}}_{3}^{-}$ registered at Los Tuxtlas was similar to that registered at other grazed sites (Table S1) and higher than the variation of ${\text{NH}}_{4}^{+}$ reported for ungrazed woodland in Australia (Yates et al., 2000). The reduction and variation of N in grazed pasture soils may be a consequence of higher microbial biomass and activity due to carbon inputs and excreta deposition by cattle that furthermore may promote net soil C and N mineralization. The low N levels may also be a result of large N losses via effects of gaseous emissions of ammonia (NH_3_), or increased leaching of ${\text{NO}}_{3}^{-}$ and/or denitrification due to high rainfall amounts at the study site.

For bulk density, pH, and organic C, similar patterns have been found for conserved sites and grazed sites in this study as on Australia sites (Paul et al., 2010a; Yates et al., 2000). Bulk density has been registered to increase under grazed pastures, whereas pH and organic C decrease (Yates et al., 2000). Variation in these soil attributes is also similar to those in Los Tuxtlas and other grazed sites (0.59%–13.30%; Table S1). This narrow range of variation did affect growth rates of some of the tree species planted (see below Effect of initial soil properties on relative growth of trees).

### 4.2 Relative growth of trees

Life history category, N_2_‐fixing capacity, and seed mass were useful to predict growth rates of species, as expected. Pioneer species outperformed nonpioneer species after six years of growth in restoration plantings. This pattern has also been reported for plantings in Brazil (eight tree species; dos Santos, Goncalves, & Feldpausch, 2006), Ecuador (15 tree species; Davidson, Gagnon, Mauffette, & Hernandez, 1998), and Mexico (14 tree species; Román‐Dañobeytia, Levy‐Tacher, Aronson, Rodrigues, & Castellanos‐Albores, 2012). Regarding N_2_‐fixing capacity, the nonpioneers legumes (*C. arborea*,* I. sinacae,* and *P. dimorphandrum*) grew as fast as the fastest pioneer species (*Ficus yoponensis*). According to the hypothesis of facultative N_2_‐fixing capacity (Barron et al., 2010), N_2_‐fixing species will outperform nonfixing species when growing under high light conditions and on soils with low N concentration; therefore, in early‐successional environments, N_2_‐fixing species may outperform nonfixing species (Batterman et al., 2013; Menge & Chazdon, 2016). This hypothesis was supported in our six‐year‐old plantings. Finally, according to other studies, seed mass is a good predictor of growth rates under high light conditions (Turnbull et al., 2012). Even when prediction strength is expected to decline after 4 years (Poorter & Rose, 2005), in our plots under managed succession, the prediction still held for the first 6 years. Furthermore, N_2_‐fixing species with large seeds showed growth rates as high as those of pioneers. In conclusion, large‐seeded N_2_‐fixing species and nonfixing species with small seeds showed high growth rates under managed succession.

### 4.3 Effect of initial soil properties on relative growth of trees

Pioneer species showed a distinct response to initial soil attributes. We found a positive response of pioneer species (*F. yoponensis* and *Cecropia obtusifolia*) to dry mass of litter, and high tolerance to high bulk density levels in mixture 1. Also, when tested individually, two pioneer species from mixture 2 (*H. appendiculatus* and *Ochroma pyramidale*) were measurably affected: They had higher relative growth rates in plots with higher pH, but notably at the lowest levels of total N and ${\text{NH}}_{4}^{+}$. The strategy of pioneer species includes a strong response to high‐resource environments, resulting in high growth rates and thus large demands of resources (King, 1994). For example, in a greenhouse experiment, fast‐growing pioneer tree species responded more to addition of N–P–K than nonpioneers (*n* = 34 tree species; Huante, Rincon, & Acosta, 1995; Huante, Rincon, & Chapin, 1998). Also, a study evaluating the response of 15 tree species to contrasting soil nutrient concentrations revealed that all three tested pioneer species responded to soil fertility (Veenendaal et al., 1996). Field experiments have shown similar results: For example, in agroforestry plantations in Kenya, fast‐growing tree species had a higher response to phosphorus addition than slow‐growing tree species (Ndufa, Shepherd, Buresh, & Jama, 1999). Also, in restoration in Mexico, nine early‐ and mid‐successional species responded to contrasting soil pH (*n* = 14 species; Roman‐Danobeytia et al., 2012). Therefore, fast‐growing pioneer species seem to respond more than slow‐growing nonpioneer species to soil attributes in early‐successional environments (but see Holl & Zahawi, 2014). On the other hand, in our study, four nonpioneer species were measurably affected by initial soil properties when tested individually: *C. arborea* (fast‐growing N_2_ fixer) and *Brosimum alicastrum* (slow growing) did grow better in plots with maximum levels of dry mass of floor litter and organic C, whereas *Tabebuia guayacan* (fast growing; Figure S2) and *Cordia megalantha* (slow growing) grew better in the plot with the highest pH (6.1). Two of these nonpioneer species showed growth rates as high as those of the pioneers planted so a strong response to resource environments is expected. Furthermore, the two slow‐growing species are considered gap‐dependent trees, i.e., canopy trees that need small gaps to reach later ontogenetic stages, but may endure shade during prolonged time as juveniles (Popma et al., 1992); therefore, these species might show slower growth rates as they were overtopped by fast‐growing pioneers, but they do respond to high‐resource environments (i.e., canopy gaps).

The response of the other seven species to initial soil properties might be concealed by N_2_‐fixing capacity or the slowest growth rates under lower light levels. For example, when N_2_‐fixing species (*P. dimorphandrum* and *I. sinacae*) were removed from the analysis of mixture 2, the remaining six nonfixing species showed a response to soil nutrient properties, benefitting from higher pH levels. In agreement with these results, in restoration plantings in Australia, three species had higher growth rates on less acidic soils (Paul, Catterall, Pollard, & Kanowski, 2010b). Furthermore, in a reforestation experiment in Tabasco, Mexico, nine tree species benefited from higher soil pH (Martínez‐Bravo, 2001). Also, in a restoration planting in Chiapas, Mexico, seven tree species had higher growth rates in soils with higher pH (7.5; Roman‐Danobeytia et al., 2012). Regarding slow growth rates, a greenhouse experiment showed that tree species with the lowest growth rates did not respond to N–P–K addition (Huante, Rincon, & Chapin, 1998). In conclusion, most of the species that responded to the range of variation in soil attributes (six of eight, Figure S2) were fast‐growing or nonfixing species.

Of the 15 tested tree species, six were promising for forest restoration on our site given high growth rates: the pioneers *F. yoponensis, C. obtusifolia,* and *H. appendiculatus* and the N_2_‐fixing nonpioneer species *C. arborea, I. sinacae,* and *P. dimorphandrum*. Apparently, relying on relatively well‐known pioneer species is a good option, when the goal is to quickly restore plant cover and make seed sources available for natural recruitment underneath (Holl & Aide, 2011). However, results showed that some nonpioneer N_2_‐fixing species may grow as fast as pioneers, and were not affected by adverse initial soil properties on our site. Small‐seeded nonpioneer species usually attain high growth rates in the open. Finally, given that dispersal limitation in degraded areas may preclude deep forest species with large seeds from colonizing restoration plantings (de la Peña‐Domene et al., 2013; Zahawi, Holl, Cole, & Reid, 2013), enrichment of plantings with large‐seeded species is suggested at a later stage to further accelerate succession.

## Conflict of Interest

None declared.
