# Supplementary material for: Effect of initial soil properties on six‐year growth of 15 tree species in tropical restoration plantings
Source: Ecol Evol. 2016 Nov 15;6(24):8686–94. doi: 10.1002/ece3.2508 (PMC5192957; doi:10.1002/ece3.2508)
Supplement: Supplementary file 1 [file ECE3-6-8686-s001.pdf]

September 22, 2016

Supplementary Material

Figure S1. Trait loading and plot scores of the PCA axes 1 and 2 of an ordination based on nine attributes for (a) eight Plantings of mixture 1, (b) eight Plantings of mixture 2 at Los Tuxtlas, Veracruz, Mexico.

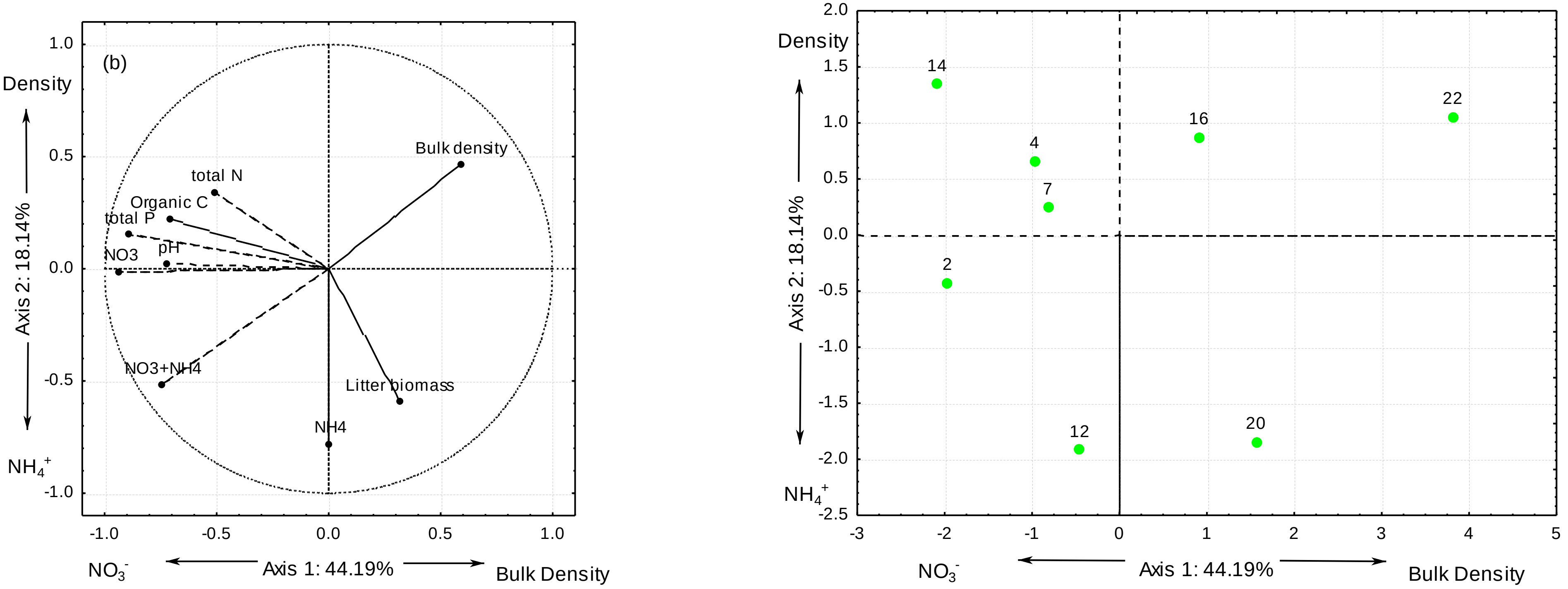

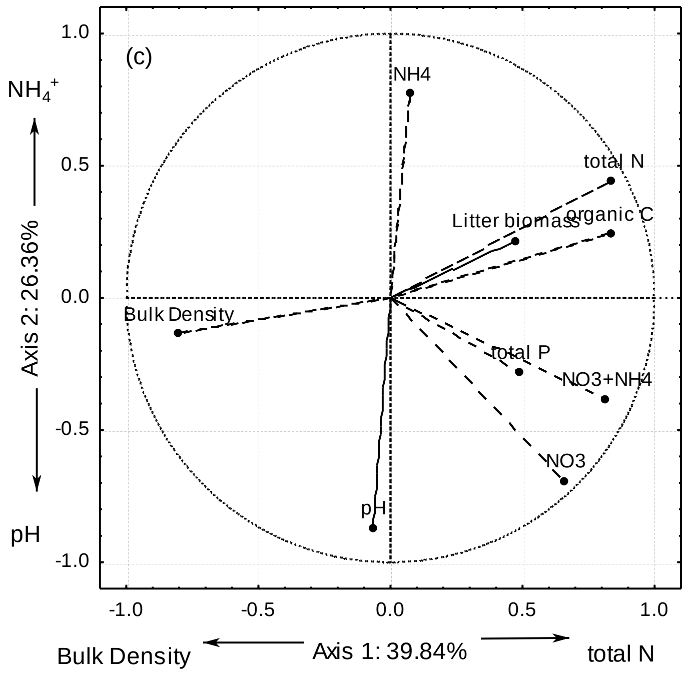

(c)
NH_4^+
pH
Axis 2: 26.36%
Axis 1: 39.84%
Bulk Density
total N
NH4
total N
Litter biomass
organic C
Bulk Density
total P
NO3+NH4
NO3
pH
1.0
0.5
0.0
-0.5
-1.0
-1.0
-0.5
0.0
0.5
1.0

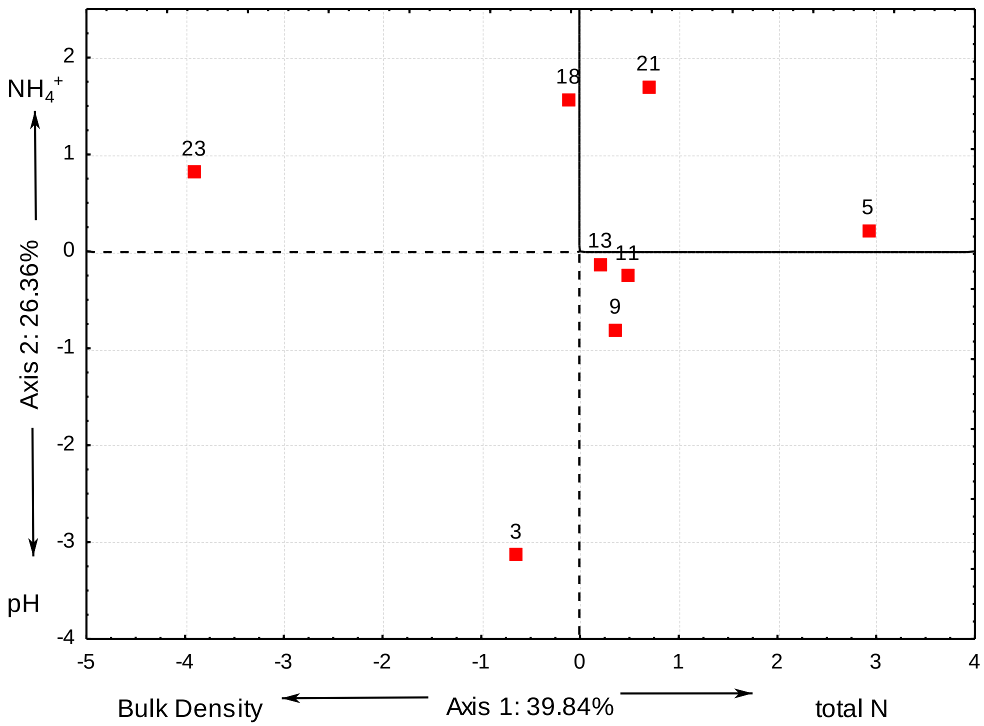

NH_4^+
pH
Axis 2: 26.36%
Axis 1: 39.84%
Bulk Density
total N
23
18
21
5
13
11
9
3
2
1
0
-1
-2
-3
-4
-5
-4
-3
-2
-1
0
1
2
3
4
