# Supplementary material for: Effect of initial soil properties on six‐year growth of 15 tree species in tropical restoration plantings
Source: Ecol Evol. 2016 Nov 15;6(24):8686–94. doi: 10.1002/ece3.2508 (PMC5192957; doi:10.1002/ece3.2508)

September 22, 2016

Supplementary Material

Figure S2. Six years *lnRGR* of diameter at the base of five pioneers (red) and 10 nonpioneer, three of them N_2-fixing tree species (green) in pastures at Los Tuxtlas, Mexico. Acronyms refer to the first five letter of the genus name. Different letters shown significant differences evaluated with Tukey test. Intermittent line separated species by growth rates: from left to right, slow growing, intermediate and fast growing tree species.

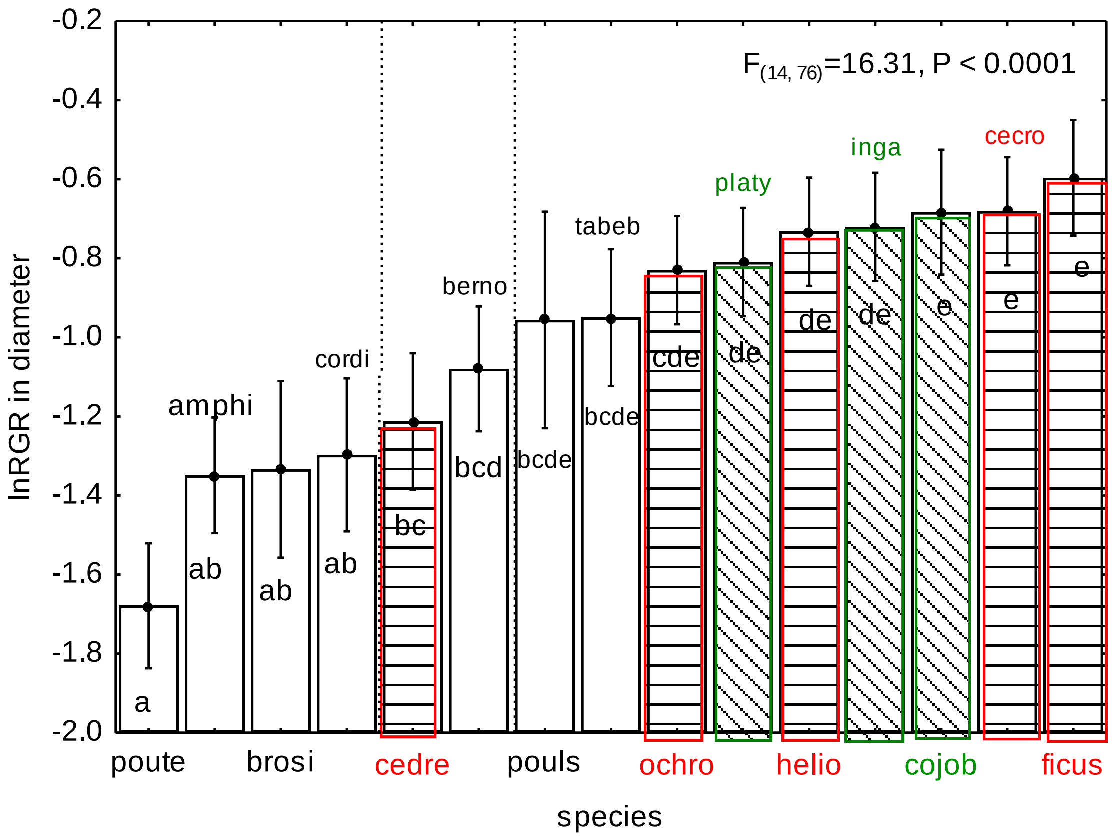

Supplement: Supplementary file 2 [file ECE3-6-8686-s002.pdf]
