# Supplementary material for: Effect of initial soil properties on six‐year growth of 15 tree species in tropical restoration plantings
Source: Ecol Evol. 2016 Nov 15;6(24):8686–94. doi: 10.1002/ece3.2508 (PMC5192957; doi:10.1002/ece3.2508)

Figure S3. Correlation between *lnRGR* in diameter at the base of 15 tree species and *ln* seed mass. Value of r^2, regression line and equation are shown. Intermittent green regression line excludes three N_2-fixing species (*lnRGR* = -1.36 - 0.10 * *ln* [Seed mass]; r^2 = 0.80; P < 0.0001) and intermittent blue regression line excludes pioneer and N_2-fixing species (*lnRGR* = -1.36 - 0.09 * *ln* [Seed mass]; r^2 = 0.75; P < 0.01). Acronyms refer to the first five letter of the genus name.

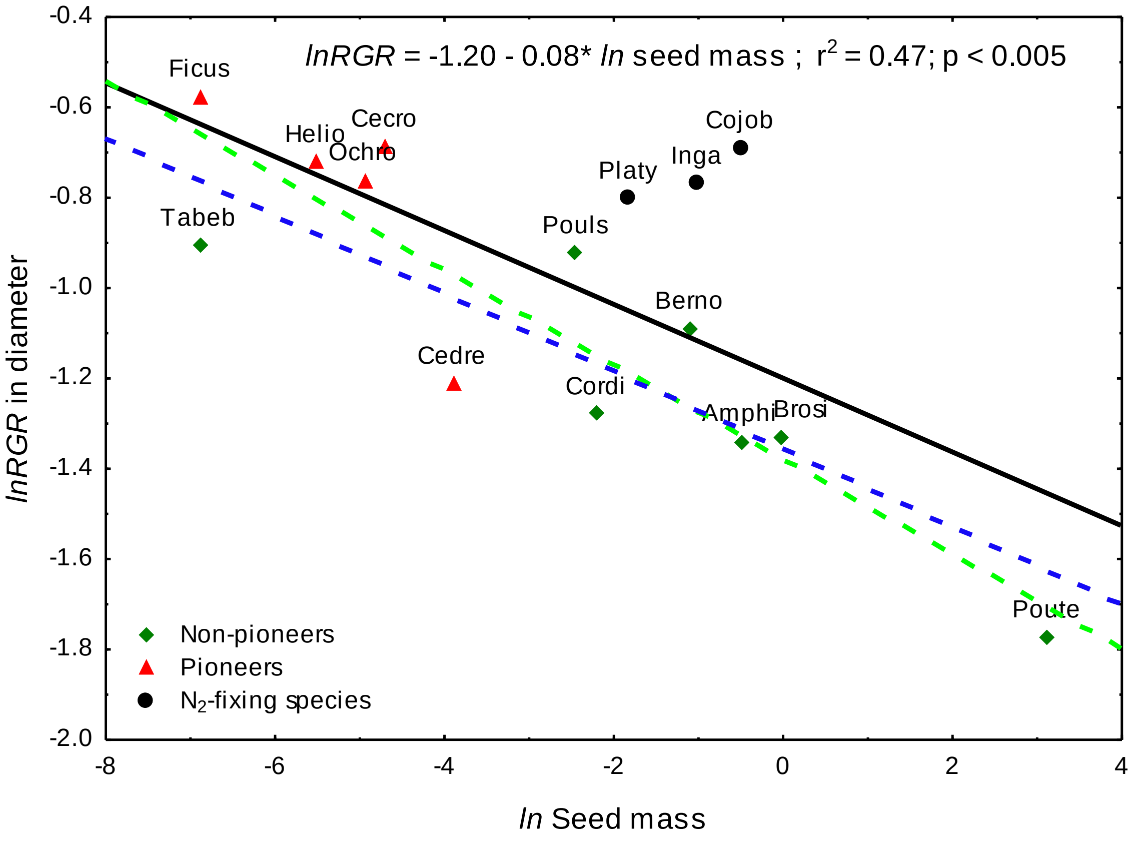

Supplement: Supplementary file 3 [file ECE3-6-8686-s003.pdf]
