# Supplementary material for: Effect of initial soil properties on six‐year growth of 15 tree species in tropical restoration plantings
Source: Ecol Evol. 2016 Nov 15;6(24):8686–94. doi: 10.1002/ece3.2508 (PMC5192957; doi:10.1002/ece3.2508)
Supplement: Supplementary file 4 [file ECE3-6-8686-s004.pdf]

Figure S4. Correlations between performance and plot PCA scores with soil attributes. PCA axes with *lnRGR* of (a) *Brosimum alicastrum*, (b) *Cojoba arborea*, (c) *Cordia megalantha*, (d) *Tabebuia guayacan*, (e) *Heliocarpus appendiculatus* and (f) *Ochroma pyramidale*. Values of r^2, regression lines and equations are shown. Arrows point out at outliers not included in regressions. In panel d intermittent regression line excludes outlier pointed with an arrow (*lnRG*R = -0.85- 0.44 * Axis 1, r^2 = 0.58). Plots were trees were growing are indicated with different symbols.

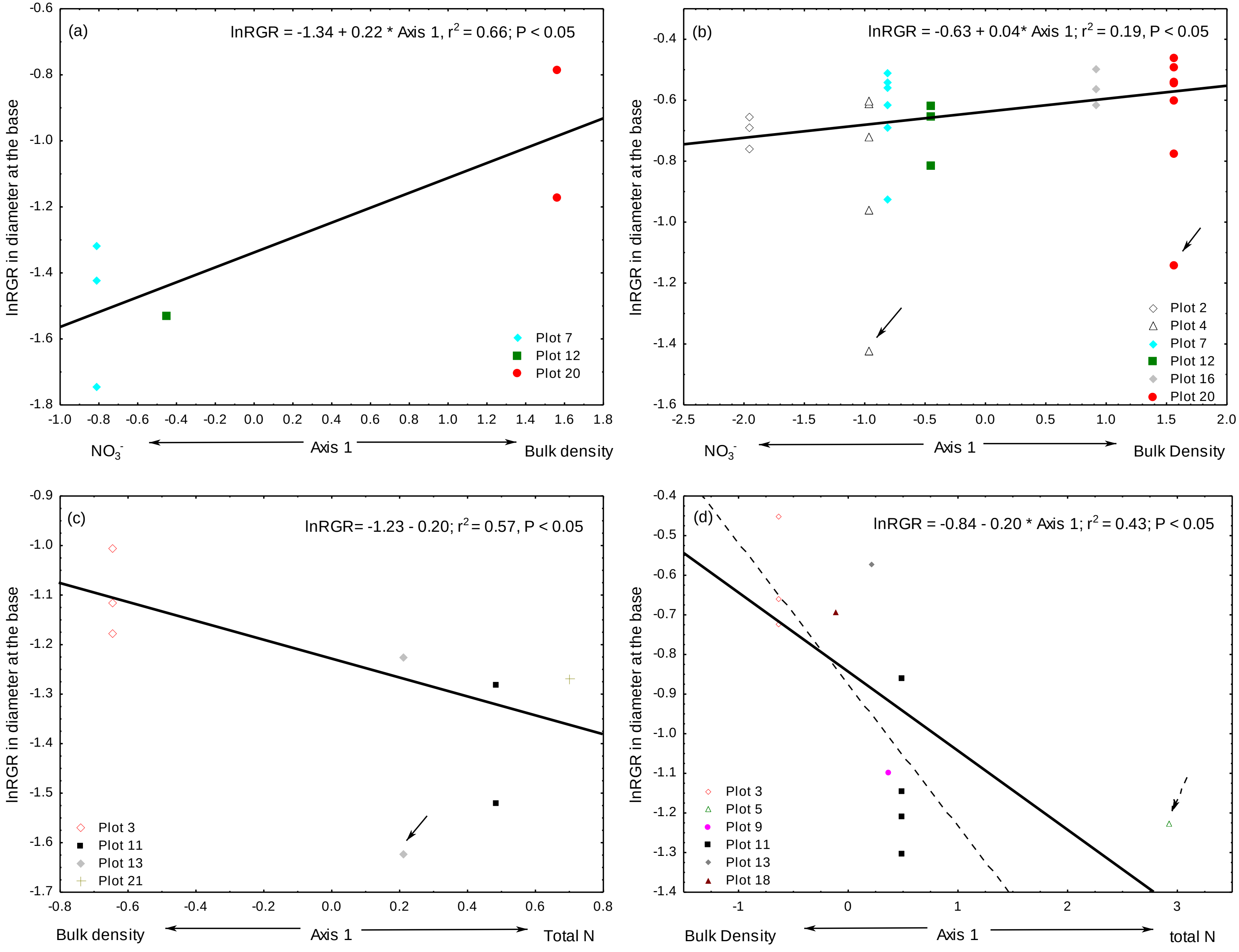

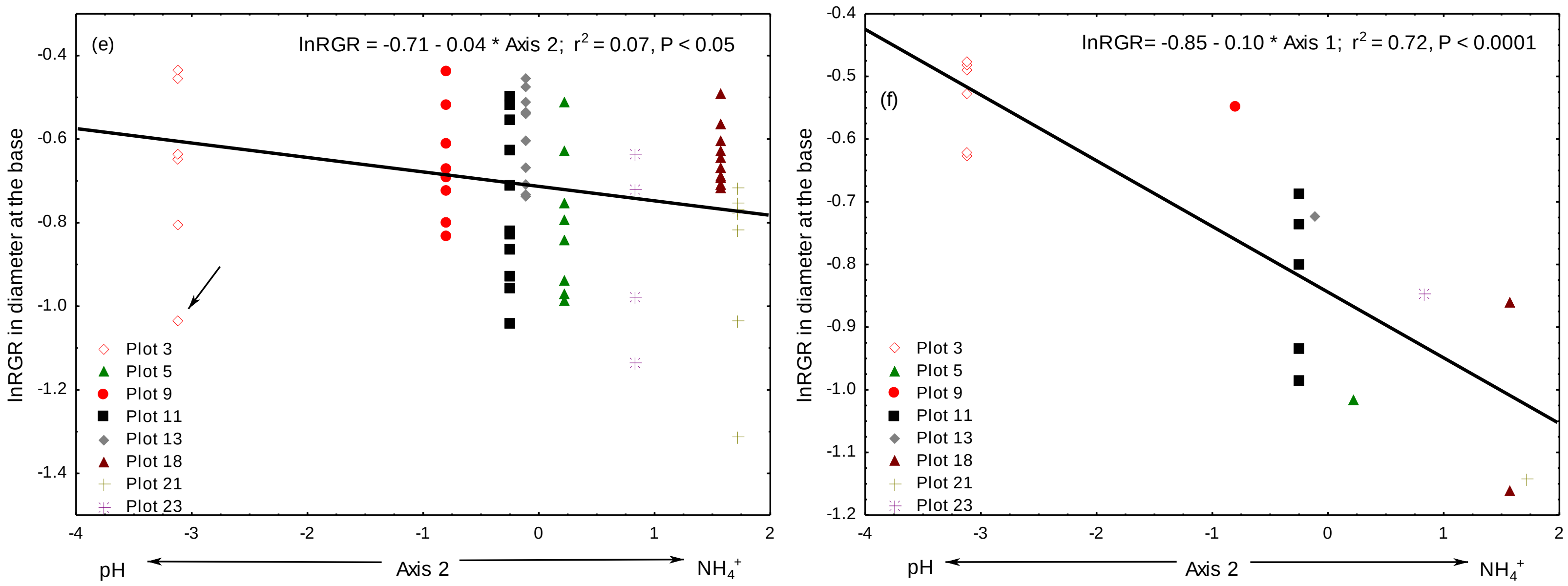
(e)
lnRGR = -0.71 - 0.04 * Axis 2; r2 = 0.07, P < 0.05
lnRGR in diameter at the base
-0.4
-0.6
-0.8
-1.0
-1.2
-1.4
Plot 3
Plot 5
Plot 9
Plot 11
Plot 13
Plot 18
Plot 21
Plot 23
-4
-3
-2
-1
0
1
2
pH
Axis 2
NH4+
(f)
lnRGR= -0.85 - 0.10 * Axis 1; r2 = 0.72, P < 0.0001
lnRGR in diameter at the base
-0.4
-0.5
-0.6
-0.7
-0.8
-0.9
-1.0
-1.1
-1.2
Plot 3
Plot 5
Plot 9
Plot 11
Plot 13
Plot 18
Plot 21
Plot 23
-4
-3
-2
-1
0
1
2
pH
Axis 2
NH4+
