# Supplementary material for: Effect of initial soil properties on six‐year growth of 15 tree species in tropical restoration plantings
Source: Ecol Evol. 2016 Nov 15;6(24):8686–94. doi: 10.1002/ece3.2508 (PMC5192957; doi:10.1002/ece3.2508)
Supplement: Supplementary file 5 [file ECE3-6-8686-s005.pdf]

Table S 1. Pearson correlation coefficients (r) of PCA axes 1 and 2 and *lnRGR* for 15 pioneer and nonpioneer species planted in 16 30 x 30 m plots in a tropical pasture at Los Tuxtlas, Veracruz, Mexico. Coefficients of determination (r^2) are shown in graphs for significant correlations.

Species	PCA axis 1	PCA axis 2
**Pioneers**		
Cecropia obtusifolia	0.22	0.20
Cedrela odorata	-0.05	-0.23
Ficus yoponensis	0.23	0.07
Heliocarpus appendiculatus	-0.05	-0.26 *
Ochroma pyramidale	-0.32	0.85**
**Non-pioneers**		
Amphitecna tuxtlensis	-0.19	0.02
Bernoullia flammea	-0.01	-0.23
Brosimum alicastrum	0.81 *	-0.54
Cojoba arborea	0.43 *	-0.11
Cordia megalantha	-0.75 *	-0.64
Inga sinacae	0.20	-0.07
Platymiscium dimorphandrum	-0.02	-0.07
Poulsenia armata	0.54	-0.54
Pouteria sapota	0.30	0.03
Tabebuia guayacan	0.76 *	0.41

*, P <0.05; **, P < 0.001

Table S2. Soil characteristics in degraded pastures and reference ecosystems in Mexico and Australia. Coefficient of variation (percentage) of soil attributes are given in parenthesis.

	Los Tuxtlas, Mexico¥		Yates et al. 2000, Australia		Paul et al. 2010, Australia	
	Rainforest	Pastures	Ungrazed woodland	Pasture	Rainforest	Pasture
Bulk density (g cm^{-3})	0.48 ± 0.08 (16.67 %)	0.95 ± 0.02 (2.10 %)	1.42 ± 0.03 (2.11 %)	1.88 ± 0.02 (1.06 %)	0.59 ± 0.08 (12.77 %)	1.11 ± 0.22 (19.03 %)
pH (H_2O)	5.95 ± 0.1 (1.68 %)	5.70 ± 0.1 (1.75 %)	7.88 ± 0.16 (2.03 %)	6.78 ± 0.04 (0.59 %)	4.93 ± 0.27 (5.53 %)	5.39 ± 0.57 (10.67 %)
Organic C (mg g^{-1})	191 ± 25.4 (13.30%)	57.4± 2.34 (4.08 %)	1.43 ± 0.08 (5.59 %)	1.14 ± 0.03§ (2.63 %)	43.4	48.8
NO_3^- (µg g^{-1})	15.1 ± 1.59 (10.53 %)	11.7 ± 0.97 (8.29 %)	4.07 ± 0.68 (16.71 %)	14.08 ± 3.5 (24.86 %)	12.69 ± 3.22 (25.04 %)	5.42 ± 2.5 (43.48 %)
NH_4^+ (µg g^{-1})	21.1 ± 4.19 (19.86 %)	6.40 ± 0.72 (11.25 %)	3.4 ± 0.16 (4.71 %)	8.2 ± 1.4 (17.07 %)	2.0 ± 1.19 (59.81 %)	4.79 ± 1.23 25.8 %)

§ (%)

¥Tobon 2009 & Tobon et al. 2011
